# Multi-omics reveals that the host-microbiome metabolism crosstalk of differential rumen bacterial enterotypes can regulate the milk protein synthesis of dairy cows

**DOI:** 10.1186/s40104-023-00862-z

**Published:** 2023-05-09

**Authors:** Chenguang Zhang, Mengya Wang, Huifeng Liu, Xingwei Jiang, Xiaodong Chen, Tao Liu, Qingyan Yin, Yue Wang, Lu Deng, Junhu Yao, Shengru Wu

**Affiliations:** College of Animal Science and Technology, Northwest A&F University, Shaanxi 712100 Yangling, China

**Keywords:** Dairy cows, Microbial and host metabolome, Milk protein, Ruminal microbiota enterotype, Structural equation model, Weighted gene co-expression network

## Abstract

**Background:**

Dairy cows’ lactation performance is the outcome of the crosstalk between ruminal microbial metabolism and host metabolism. However, it is still unclear to what extent the rumen microbiome and its metabolites, as well as the host metabolism, contribute to regulating the milk protein yield (MPY).

**Methods:**

The rumen fluid, serum and milk of 12 Holstein cows with the same diet (45% coarseness ratio), parity (2–3 fetuses) and lactation days (120–150 d) were used for the microbiome and metabolome analysis. Rumen metabolism (rumen metabolome) and host metabolism (blood and milk metabolome) were connected using a weighted gene co-expression network (WGCNA) and the structural equation model (SEM) analyses.

**Results:**

Two different ruminal enterotypes, with abundant *Prevotella* and *Ruminococcus*, were identified as type1 and type2. Of these, a higher MPY was found in cows with ruminal type2. Interestingly, *[Ruminococcus] gauvreauii* group and *norank_f_Ruminococcaceae* (the differential bacteria) were the hub genera of the network. In addition, differential ruminal, serum and milk metabolome between enterotypes were identified, where the cows with type2 had higher *L*-tyrosine of rumen, ornithine and *L*-tryptophan of serum, and tetrahydroneopterin, palmitoyl-*L*-carnitine, *S*-lactoylglutathione of milk, which could provide more energy and substrate for MPY. Further, based on the identified modules of ruminal microbiome, as well as ruminal serum and milk metabolome using WGCNA, the SEM analysis indicated that the key ruminal microbial module1, which contains the hub genera of the network (*[Ruminococcus] gauvreauii* group and *norank_f_Ruminococcaceae*) and high abundance of bacteria (*Prevotella* and *Ruminococcus*), could regulate the MPY by module7 of rumen, module2 of blood, and module7 of milk, which contained *L*-tyrosine and *L*-tryptophan. Therefore, in order to more clearly reveal the process of rumen bacterial regulation of MPY, we established the path of SEM based on the *L*-tyrosine, *L*-tryptophan and related components. The SEM based on the metabolites suggested that *[Ruminococcus] gauvreauii* group could inhibit the energy supply of serum tryptophan to MPY by milk *S*-lactoylglutathione, which could enhance pyruvate metabolism. *Norank_f_Ruminococcaceae *could increase the ruminal *L*-tyrosine, which could provide the substrate for MPY.

**Conclusion:**

Our results indicated that the represented enterotype genera of *Prevotella* and *Ruminococcus*, and the hub genera of *[Ruminococcus] gauvreauii* group and *norank_f_Ruminococcaceae* could regulate milk protein synthesis by affecting the ruminal *L*-tyrosine and *L*-tryptophan. Moreover, the combined analysis of enterotype, WGCNA and SEM could be used to connect rumen microbial metabolism with host metabolism, which provides a fundamental understanding of the crosstalk between host and microorganisms in regulating the synthesis of milk composition.

**Supplementary Information:**

The online version contains supplementary material available at 10.1186/s40104-023-00862-z.

## Introduction

As the world population and demand for high-quality animal protein continue to increase, dairy milk has become an indispensable high-nutritional animal protein product [1]. Rumen microbial digestion and metabolism provide energy and precursors for milk composition synthesis in dairy cows [2]. It has been proven that compared to the rumen microbiome of low milk protein yield (MPY) cows, the rumen microbial KEGG function of high MPY cows enriched in the pyruvate metabolism and reduced in the methane metabolism [3]. Further, the methane emission and feed efficiency were reported to be affected by ruminal microbiome and metabolome [4, 5]. The milk protein biosynthesis in dairy cows is a complicated biological process that involves not only the rumen, but also host metabolic processes [3]. The milk protein biosynthesis could be briefly affected by several biological processes of ruminal dietary crude protein degradation, ruminal microbial protein and amino acid synthesis, intestinal dietary protein and microbial protein degradation, intestinal digested and microorganism synthesized amino acid absorption, and hepatic and mammary gland amino acid metabolism and biotransformation [6]. Hence, when focusing overall on the metabolome changes from rumen-blood-mammary gland axis insight, the roles of key bacteria and key bacteria-driven ruminal microbiome in regulating the milk protein synthesis-related metabolism pathways were still insufficient.

Reproducible patterns of variation in the microbiota, like the major proportions such as *Bacteroides* and *Prevotella*, have been observed in the human gut [7]. When separated into different clusters, they have been identified as the “enterotypes” [7] and proposed as a useful method to stratify human gut microbiomes. Later, other studies found stratification in other ecosystems, such as the vagina [8] and other body sites [9, 10]. By revisiting the enterotype concept, three enterotypes of humans, which were separately driven by *Bacteroides*, *Prevotella*, and *Ruminococcus* were identified [11, 12]. An investigation of the properties of each enterotype revealed networks of co-occurring microbes centered around the indicator (driver) genera as well, which could be linked to phenotype changes, such as the body weight of humans [11, 12]. Hence, we presume that enterotype analysis can help in identifying key bacteria and link the key bacteria driven ruminal microbiome to the milk protein synthesising ability.

In order to systemically analyze the metabolome changes of rumen-blood-mammary gland axis and identify the contribution of ruminal microbiome and metabolome to the milk protein synthesis, clustering analyses were performed using the weighted gene co-expression network (WGCNA) and the structural equation model (SEM) analyses. These could help to link the ruminal microbiome as well as the ruminal, serum, and milk metabolome, and identify the pathway that ruminal microbiome affected in the milk protein synthesis by changing the microbial and host metabolome. Therefore, we grouped the dairy cows according to ruminal enterotypes and explored the relationship between enterotypes and MPY by analysing the overall ruminal microbiome, as well as ruminal, serum, and milk metabolome, using a combined analysis of WGCNA and SEM.

## Materials and methods

### Ethics approval statement

This experiment was conducted at the Animal Research and Technology Centre of Northwest A&F University (Yangling, Shaanxi, China). It was performed in accordance with the guidelines recommended by the Administration of Affairs Concerning Experimental Animals (Ministry of Science and Technology, China, revised 2004). The protocol was approved by the Institutional Animal Care and Use Committee at Northwest A&F University.

### Animal, study design, and sample collection

A cohort of 871 healthy lactating Holstein cows housed at a commercial dairy farm in Ning Xia, China. Of these, the 12 lactating Holstein cows involved in the study were randomly selected from the 97 Holstein cows with the same parity (2^nd^ litter), similar lactation days (120–150 d), and similar body condition (body condition score from 2.5 to 3). The 12 selected cows were raised from early to middle lactation (21–200 d after calving). The animals were given the same feedlot diet with a 45% coarseness ratio (dry matter basis). All the cows were fed and milked thrice a day at 06:00, 14:00 and 22:00, and were given free access to water and feed. The daily milk quality and dairy yield of these 12 selected lactating Holstein cows were analysed. Briefly, the feed (including the alfalfa and starter feed) offered was adjusted daily to ensure at least 10% orts. The feed intake of the cows was recorded for three consecutive days by artificially recording the initial feed weight and the weight of the remaining material after each free intake of each cow.

In this study, the milk samples of the 12 cows were collected during the lactation period of 130–150 d. The milk yield (MY), MPY, milk fat yield (MFY), and lactose of the 12 dairy cows were recorded as 29.64 ± 0.290 kg/d, 1.16 ± 0.050 kg/d, 1.12 ± 0.016 kg/d, and 1.46 ± 0.023 kg/d (mean ± standard error of the mean) respectively. During the experimental sampling period (lactation period of 130–150 d), the rumen fluid was sampled using oral stomach tubes and filtered through four layers of cheesecloth, and then used for 16S rRNA gene sequencing and metabolome analysis. Blood samples from all dairy cows were collected in tubes without anticoagulants. Then, serum samples were separated by centrifugation at 3,500 × *g* for 15 min at 4 °C (using Centrifuge 5810R, Eppendorf, Germany) to measure the chemical parameters and metabolome in the serum. The milk was sampled for milk quality detection and metabolome analysis.

### Determination of milk composition

The fat, protein, and lactose contents in the milk were measured using infrared analysis through a spectrophotometer (Foss-4000; Foss Electric A/S, Hillerød, Denmark).

### Determination of volatile fatty acids (VFA) concentrations in ruminal fluid

The rumen fluid samples were centrifuged at 13,000 × *g* for 10 min at 4 °C. The supernatant samples were analysed for volatile fatty acids (VFA) concentration using an Agilent 6850 gas chromatograph (Agilent Technologies Inc., Santa Clara, CA, USA) equipped with a polar capillary column (HP-FFAP, 30 m × 0.25 mm, 0.25 μm) and a flame ionisation detector, as previously described [13, 14].

### Determination of serum biochemical metabolites composition

The total protein (TP), glucose (GLU), total cholesterol (TC), and triglyceride (TG) were determined using commercial kits as per the manufacturer’s instructions (Beijing Huaying Co., Ltd., Beijing, China).

### DNA extraction

The total genomic DNA was extracted from rumen contents using the repeat bead-beating plus column method [15]. Nuclease-free water was used for the blank. The final DNA concentration and purification were determined through fluorometry using a Qubit 2.0 fluorometer (Life Technologies, Grand Island, NY, USA). The total DNA was eluted in 50 μL of elution buffer and stored in a −80 °C freezer until further library preparation and sequencing.

### Microbiota 16S rRNA gene sequencing, analysis and identification of rumen bacterial enterotypes

The V3–V4 regions of the 16S rRNA genes were amplified with Illumina sequencing index-binding primer pairs 338F (5'-ACTCCTACGGGAGGCAGCAG-3') and 806R (5'-GGACTACHVGGGTWTCTAAT-3') [16] in the following PCR conditions: 30 s at 95 °C, 30 s at 55 °C, and 45 s at 72 °C for 27 cycles. PCRs were performed using 4 μL 5 × TransStart FastPfu buffer, 2 μL 2.5 mmol/L deoxynucleoside triphosphates (dNTPs), 0.8 μL of each primer (5 μmol/L), 0.4 μL TransStart FastPfu DNA polymerase, 10 ng of extracted DNA, and extra ddH_2_O in a 20-μL system. Agarose gel electrophoresis was performed to verify the size of amplicons. The completed libraries were quantified using Quant-iT fluorometric assay (Thermo Fischer Scientific, Waltham, MA, USA). Two of 48 sample libraries with concentrations less than 2 nmol/L were discarded. Thereafter, paired-end sequences (2 × 300 bp) of the remaining 46 prepared sample libraries were generated on an Illumina MiSeq sequencing platform (Illumina, San Diego, CA, USA), using MiSeq Reagent Kit v3 (Illumina).

Raw FASTQ files were de-multiplexed using an in-house perl script, and then quality-filtered by fastp version 0.19.6 [17] and merged by FLASH version 1.2.7 [18] with the following criteria: (i) the 300 bp reads were truncated at any site receiving an average quality score of < 20 over a 50 bp sliding window, and the truncated reads shorter than 50 bp were discarded, reads containing ambiguous characters were also discarded; (ii) only overlapping sequences longer than 10 bp were assembled according to their overlapped sequence, the maximum mismatch ratio of overlap region is 0.2, and reads that could not be assembled were discarded; (iii) samples were distinguished according to the barcode and primers, and the sequence direction was adjusted, exact barcode matching, 2 nucleotide mismatch in primer matching. To minimize the effects of sequencing depth on alpha and beta diversity measures, the number of sequences from each sample was rarefied to 28,788 (the lowest read). Then the high-quality sequences were de-noised and the amplicon sequence variants (ASVs) was assembled using DADA2 [19] in the QIIME2 [20] pipeline under default parameters, which gave single nucleotide resolution based on error profiles within samples. Finally, 693 ASVs per sample were used to rarefaction and downstream analysis. Taxonomic assignment of ASVs was performed using the Naive Bayes consensus taxonomy classifier implemented in QIIME2 and the SILVA 16S rRNA database (v138, https://www.arb-silva.de/silva-license-information/) [21].

The following analysis on alpha and beta diversity was performed on the filtered data (rarefied abundance table) using USEARCH alpha_div [22] and UniFrac metrics [23] in QIIME2, respectively. ASV richness estimates (Chao 1, Abundance-based Coverage Estimator: ACE) and diversity indices (Simpson) were used to measure microbiota alpha diversity in all the samples. Beta diversity from different samples were compared via PCoA analysis based on Bray-Curtis distance matrices.

Partitioning Around Medoids (PAM) clustering was performed based on the Jensen-Shannon divergence (JSD). The best clustering K number was calculated using the Calinski-Harabasz (CH) index [7]. The ruminal bacterial enterotypes were analysed using between-class analysis (BCA) [7].

### Construction of the genera interaction network

Network graphs were calculated based on the correlation of the abundance of all the tested genera using the R package ggClusterNet [24], which could complete the whole microbiome and bipartite network analysis from correlations calculation, network visualisation, network properties calculation, and node properties and construction of the random networks and comparation. Based on the network properties calculation and node properties, the key genera of the network were identified. The genera with high igraph.degree value in the network graph are identified as key genera.

### Shotgun metagenome sequencing and data processing

The same DNA samples were used for metagenome sequencing. The DNA extracts were fragmented to an average size of about 400 bp using Covaris M220 (Gene Company Limited, China) for paired-end library construction. Paired-end library was constructed using NEXTFLEX Rapid DNA-Seq (Bioo Scientific, Austin, TX, USA). Adapters containing the full complement of sequencing primer hybridisation sites were ligated to the blunt end of fragments. Paired-end sequencing was performed on Illumina NovaSeq/Hiseq Xten(Illumina Inc., San Diego, CA, USA) using NovaSeq Reagent Kits/HiSeq X Reagent Kits, according to the manufacturer’s instructions (www.illumina.com).

The quality control of each dataset was performed using Sickle (version 1.33, https://github.com/najoshi/sickle) to trim the 3’-end of reads and 5’-end of reads, cut low-quality bases (quality scores < 20), and remove short reads (< 50 bp) and “N” records. The reads were aligned to the bovine genome (bosTau8 3.7, https://doi.org/10.18129/B9.bioc.BSgenome.Btaurus.UCSC.bosTau8) using BWA (http://bio-bwa.sourceforge.net) to filter out the host DNA [25]. The filtered reads were de novo assembled for each sample using Megahit (https://github.com/voutcn/megahit) [26]. MetaGene (http://metagene.cb.k.u-tokyo.ac.jp/) was used to predict open reading frames (ORFs) from assembled contigs of length > 300 bp [27]. The assembled contigs were then pooled and non-redundancies were constructed based on the identical contigs using CD-HIT with 95% identity (http://www.bioinformatics.org/cd-hit/) [27]. The original sequences were mapped to the predicted genes and the abundances were estimated using SOAPaligner (http://soap.genomics.org.cn/) [28].

The contigs were annotated using DIAMOND against the KEGG database (http://www.genome.jp/kegg/), with an E value of 1e−5 [29]. Abundances of the KEGG pathway were normalised into Trans Per Million reads (TPM) for downstream analysis. KEGG pathways with TPM > 5 in at least 50% of the animals within each group were used for the downstream analysis.

### Metabolomic analysis

The rumen fluid, serum and milk samples from the 12 cows were used for metabolomic analysis. Approximately 100 μL of rumen fluid and milk samples were preprocessed for metabolomic analyses. All sample scans were acquired using the LC-MS system, following the manufacturer’s instructions. Briefly, the metabolites were extracted using 400 µL methanol:water (4:1, v/v) solution. The mixture was allowed to settle at −20 °C and treated using a high-throughput tissue crusher, Wonbio-96c (Shanghai Major biotechnology Co., LTD), at 50 Hz for 6 min, followed by vortex for 30 s and ultrasound at 40 kHz for 30 min at 5 °C. The samples were placed at −20 °C for 30 min to precipitate proteins. After centrifugation at 13,000 ×  at 4 °C for 15 min, the supernatant was carefully transferred to sample vials for LC-MS/MS analysis. Meanwhile, as part of the system conditioning and quality control process, a pooled quality control sample (QC) was prepared by mixing equal volumes of all samples. The QC samples were disposed of and tested in the same manner as the analytic samples.

Chromatographic separation of the metabolites was performed on an ExionLC^TM^ AD system (AB Sciex, Framingham, MA, USA) equipped with an Acquity UPLC BEH C18 column (100 mm × 2.1 mm i.d., 1.7 µm; Waters, Milford, USA). The mobile phases consisted of 0.1% formic acid in water with formic acid (0.1%) (solvent A) and 0.1% formic acid in acetonitrile:isopropanol (1:1, v/v) (solvent B). The solvent gradient changed according to the following conditions: from 0 to 3 min, 95% (A):5% (B) to 80% (A):20% (B); from 3 to 9 min, 80% (A):20% (B) to 5% (A):95% (B); from 9 to 13 min, 5% (A):95% (B) to 5% (A):95% (B); from 13 to 13.1 min, 5% (A):95% (B) to 95% (A):5% (B), from 13.1 to 16 min, 95% (A):5% (B) to 95% (A):5% (B) for equilibrating the systems. The sample injection volume was 20 μL and the flow rate was set to 0.4 mL/min. The column temperature was maintained at 40 °C. During the period of analysis, all these samples were stored at 4 °C.

The UPLC system was coupled with a quadrupole-time-of-flight mass spectrometer (Triple TOF^TM^ 5600 + , AB Sciex) equipped with an electrospray ionisation (ESI) source operating in positive and negative modes. The optimal conditions were set as followed: source temperature, 500 °C; curtain gas (CUR), 30 psi; Ion Source GS1 and GS2, 50 psi; ion-spray voltage floating (ISVF), −4000 V in negative mode and 5000 V in positive mode, respectively; declustering potential, 80 V; a collision energy (CE), 20–60 V rolling for MS/MS. Data acquisition was performed in the Data Dependent Acquisition (DDA) mode. The detection was carried out over a mass range of 50–1000 *m/z*.

After UPLC-TOF/MS analyses, the raw data were imported into Progenesis QI 2.3 (Nonlinear Dynamics, Waters, USA) for peak detection and alignment. The preprocessing results generated a data matrix that consisted of the retention time (RT), mass-to-charge ratio (*m/z*) values, and peak intensity. Any set of samples in which at least 80% metabolic features were detected were retained. After filtering, minimum metabolite values were imputed for specific samples in which the metabolite levels fell below the lower limit of quantitation and each metabolic features were normalised using sum. The internal standard was used for data QC (reproducibility). Metabolic features for which the relative standard deviation (RSD) of QC > 30% were discarded. Following normalisation procedures and imputation, statistical analysis was performed on log-transformed data to identify significant differences in metabolite levels between comparable groups. Mass spectra of these metabolic features were identified by using accurate mass. MS/MS fragments spectra and isotope ratio difference were searched on reliable biochemical databases such as Human Metabolome Database (HMDB) (http://www.hmdb.ca/) and Metlin database (https://metlin.scripps.edu/). Concretely, the mass tolerance between the measured *m/z* values and the exact mass of the components of interest was ±10 ppm. For metabolites having MS/MS confirmation, only the ones with MS/MS fragments score above 30 were considered as confidently identified. Otherwise, metabolites were given only tentative assignments.

The analysis methods used were principal component analysis (PCA) and orthogonal partial least-squares discriminant analysis (OPLS-DA). Supervised OPLS-DA was conducted through metaX [30] to discriminate the different variables between groups. The variable important for the projection (VIP) value was calculated, and a VIP cut-off value of 1.0 was used to select important features (VIP ≥ 1; ratio ≥ 2 or ratio ≤ 1/2; *q* value ≤ 0.05).

Differential metabolites were summarised and mapped into their biochemical pathways through metabolic enrichment and pathway analysis, based on the KEGG database (http://www.genome.jp/kegg/). The scipy.stats (Python packages) ( https://docs.scipy.org/doc/scipy/) was exploited to identify statistically significantly enriched pathways using Fisher’s exact test.

### Weighted gene co-expression network analysis (WGCNA)

WGCNA was used to identify key phenotype-related metagenomic and metabolic modules based on correlation patterns. WGCNA was performed using R packages WGCNA [31] and vegan [32], after going through official tutorials (https://horvath.genetics.ucla.edu). To describe the MGB metabolic network features comprehensively, we integrated peripheral and central metabolites into a scale-free network topology, and normalised the abundance with logarithmic conversion and robust quantile normalisation. We used a ‘step-by-step network construction’ for metabolic network topology, adjusted network type to a ‘signed hybrid’ and set the soft thresholding power to 7 (rumen microbiome, Additional file 1: Fig. S1A), 18 (rumen metabolome, Additional file 1: Fig. S1B), 16 (blood metabolome, Additional file 1: Fig. S1C) and 20 (milk metabolome, Additional file 1: Fig. S1D) to obtain the best topological overlap matrix, and kept other parameters as default. Based on the distance matrix, genes were subsequently clustered using the average linkage hierarchical clustering method using hclust, and the expression modules were detected using dynamicTreeCut. Modules with similar patterns were further clustered and merged into consensus modules. The correlation between the consensus modules and milk composition was calculated using corPvalueStudent. Pairwise Pearson correlation coefficients were calculated for all the selected genes. The resulting Pearson correlation matrix was transformed into a matrix of connection strengths (an adjacency matrix) using a power function, which was then converted into a topological overlap matrix. WGCNA seeks to identify modules of densely interconnected genes using hierarchical clustering based on topological overlap.

### Structural equation modelling construction (SEM) analysis

SEM was constructed to evaluate the direct link among rumen microbiome modules, rumen metabolome modules, serum metabolome, milk metabolome modules, and milk compositions, as well as among identified differential genera, ruminal, serum, and milk differential metabolites. The goodness-of-fit of the SEM was checked using the χ^2^ test, the root mean square error (RMSE), and the comparative fit index (CFI). The model had a good fit when the CFI value was close to 1 and the *P* values of the statistics were high (traditionally, > 0.05) [33]. SEM was conducted using the lavaan package [34].

### Statistics

The statistical analyses were performed using the “stats” package in R (https://www.r-project.org) [35]. The homogenized microbial abundance (relative abundance) was used for subsequent analysis. The Mann-Whitney U test with multiple comparisons adjusted by the Benjamini-Hochberg FDR was performed to compare the microbial alpha diversity and the different bacteria of 16S rRNA gene sequencing results between two groups (*P* < 0.05). ANOSIM analysis based on Bray-Curtis distance matrices was used to identify the beta diversity between two or more compared groups. The one-way analysis of variance (ANOVA) was performed to compared to the different lactating performance (milk yield, fat, protein, and lactose) between groups (*P* < 0.05). Pairwise correlations of network in ggClusterNet were calculated using Spearman’s correlation, and *P* < 0.05 and Spearman’s correlation indices > 0.5 were used to generate all significant relationships in the present study.

## Results

### Identification of ruminal microbiota enterotypes, and microbial and phenotypes features of differential enterotypes

**Fig. 1:**
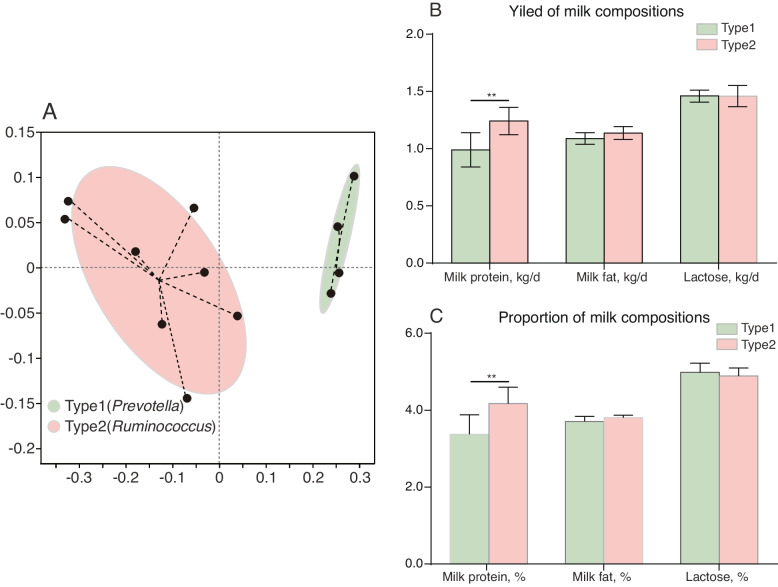
12 cows were grouped according to ruminal enterotype. **A** The enterotype grouping for the rumen fluid samples of 12 cows using 16S rRNA sequence data based on the Bray-Curtis distance. **B** The differences of milk composition yield between enterotypes. **C** The differences of milk composition proportion between enterotypes. ^**^P < 0.01

In order to establish the relationship between rumen bacteria and lactating performance under the same feeding and management, the rumen microbiome was divided into type1 (*n* = 4) and type2 (*n* = 8) using enterotype identification (Fig. 1A). No significant difference of dry matter intake was identified (21.21 ± 0.82 vs. 20.37 ± 0.86 kg/d). Next, the lactating performances of cows in the type1 and type2 groups were compared. The MPY and milk protein composition of cows in the type2 ruminal microbiota enterotype were found to be higher than that of the cows in type1 (*P* = 0.044), while the other lactation performances had not significantly changed (Fig. 1B). Further, only the serum total protein content tended to increase (*P* = 0.054) in cows with type2 ruminal microbiota enterotypes; the ruminal VFA and serum glucose, total cholesterol, and triglyceride had not changed between different enterotypes (Tables 1 and 2). Herein, we supposed that these 12 dairy cows, with similar lactation yield under the same dietary, parity, and lactation phases, provide a suitable model to illuminate the ruminal microbiota enterotypes’ roles in regulating MPY.

**Table 1:** Rumen volatile fatty acids of type1 and type2 cows

Items^a^	Mean		SEM^b^	*P*-value
	Type1	Type2		
Acetate, mol/L	81.96	84.56	3.537	0.736
Propionate, mol/L	34.46	33.14	1.962	0.757
Isobutyrate, mol/L	0.93	1.04	0.0629	0.397
Butyrate, mol/L	15.22	15.49	0.864	0.884
Isovalerate, mol/L	1.60	1.84	0.114	0.299
Valerate, mol/L	2.30	2.28	0.117	0.937
TVFA, mol/L	136.47	138.36	6.175	0.889
A/P	2.43	2.57	0.0816	0.426

^a^*TVFA* Total volatile fatty acids, *A/P* Acetate/ Propionate

^b^*SEM* Standard error mean

**Table 2:** Serum biochemical level of type1 and type2 cows

Items^a^	Mean		SEM^b^	*P*-value
	Type1	Type2		
TP, g/L	78.66	83.075	2.276	0.054
GLU, mmol/L	1.90	2.39	0.245	0.217
TC, mmol/L	5.32	6.18	0.410	0.345
TG, mmol/L	0.14	0.13	0.0116	0.960

^a^*TP* Total protein, *GLU* Glucose, *TC* Total cholesterol, *TG* Triglyceride

^b^*SEM* Standard error mean

**Fig. 2:**
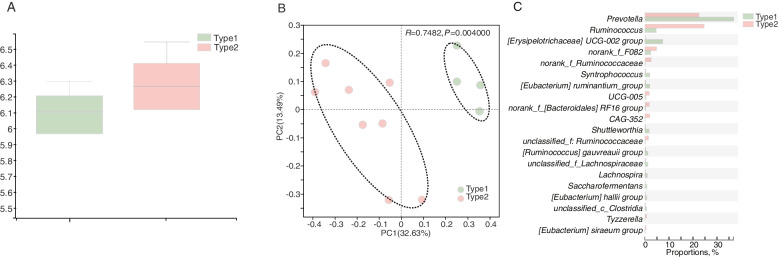
Ruminal bacterial compositional profiles of type1 and type2 cows. **A** The α-diversity between type1 and type2 using Shannon index. **B** Bacterial compositional profiles of type1 and type2 rumen samples based on species visualised using principal-coordinate analysis (PCoA). **C** Differential bacterial compositions (gene level) using 16S rRNA sequence data based on Wilcoxon rank sum test

For α-diversity, no difference was identified between the Shannon index of cows with type1 and type2 enterotypes (Fig. 2A). Obviously, the rumen microbiome of type1 and type2 enterotypes showed a significant distinct clustering in the PCoA plot (ANOSIM: *R* = 0.74, *P* = 0.004) (Fig. 2B). With respect to microbial compositions, the most abundant genera presented in the type1 and type2 enterotypes were *Prevotella* and *Ruminococcus* respectively (Additional file 1: Fig. S2). Differential bacteria were also observed (Fig. 2C and Additional file 2: Table S1). *Prevotella*, [*Erysipelotrichaceae] UCG-002* group, *Syntrophococcus*, *[Eubacterium] ruminantium *group, *Shuttleworthia*, *Ruminococcus gauvreauii *group, *unclassified_f*__*Lachnospiraceae*, *Lachnospira*, *Saccharofermentans*, *[Eubacterium] hallii *group, and *unclassified_c*__*Clostridia* were higher in type1 (*P* < 0.05), while *Ruminococcus*, *norank_f*__*F082*, *norank_f*__*Ruminococcaceae*, *UCG-005*, *norank_f*__*Bacteroidales_RF16 *group, *CAG-352 *group, *unclassified_f*__*Ruminococcaceae*, *Tyzzerella*, *[Eubacterium] siraeum *group, *[Erysipelotrichaceae] UCG-002 *group, and *norank_f*__*UCG-010* were higher in type2 (*P* < 0.05).

### Identification of the core genera of microbial network and the related microbial functions

**Fig. 3:**
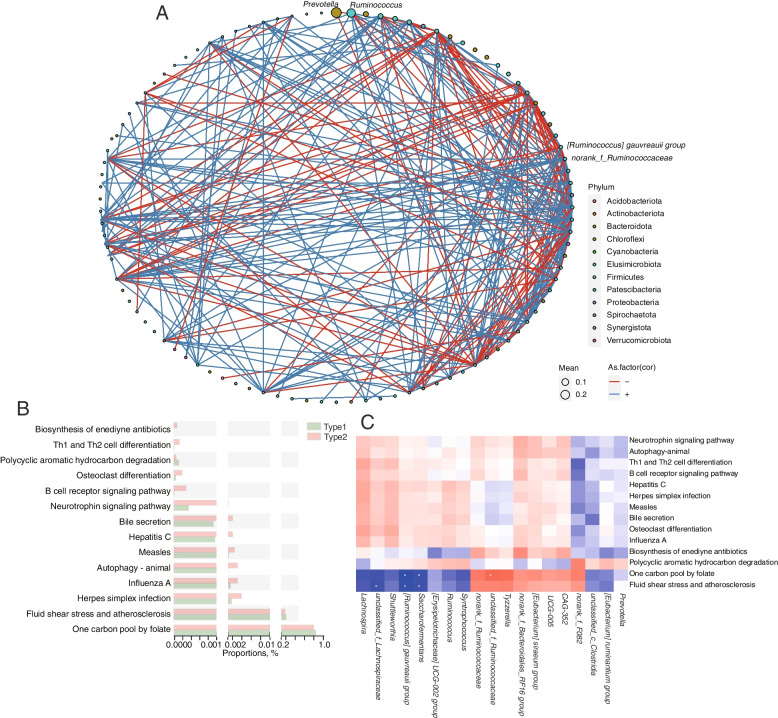
Differential bacterial compositions and functions between type1 and type2 cows. **A** Network analysis of all dairy cows. **B** Differential bacterial functions using metagenome data based on Wilcoxon rank sum test. **C** Correlation analysis between differential bacterial compositions and functions

Although *Prevotella* and *Ruminococcus* were the representative bacteria in the enterotypes, they were not the core bacteria in the microbiome network established by the 12 cows. *[Ruminococcus] gauvreauii *group and *norank_f*_*Ruminococcaceae*, which were also the significantly differential genera between the enterotypes, were identified as the hub genera of the network according to the identified betweenness and degree (Fig. 3A and Additional file 2: Table S2). Moreover, the microbiome networks established by the four cows of type1 (Additional file 1: Fig. S3A and Additional file 2: Table S3) and the eight cows of type2 (Additional file 1: Fig. S3B and Additional file 2: Table S4) were also identified. *Ruminococcus* and *Prevotella* were identified as the hub genera of type1 enterotype, while *norank__f*__*Ruminococcaceae* and *[Ruminococcus] gauvreauii *group were identified as the hub genera of type2 enterotype. As the hub genera, we also provided the ASVs sequence of *norank_f_Ruminoccous* (Additional file 2: Table S17). Notably, when compared with the type2 enterotypes, the genera network of type1 was found to have more crosstalk among identified genera.

In order to further investigate the effect of the enterotypes on the rumen microbial function, metagenome sequencing was performed. A total of 592,766,308 reads, with 53,667,000 ± 2,048,010 reads (mean ± SD) per sample were generated. The microbial functions of the enterotypes were determined using genomes (KEGG) profiles. The “one carbon pool by folate” and “fluid shear stress and atherosclerosis” profiles were found to be significantly enriched and increased in the type1 enterotypes (*P* < 0.05) (Fig. 3B). Next, the correlation analysis between differential genera and differential KEGG pathway (level3) was performed (Fig. 3C). The “one carbon pool by folate” profile was negatively related to *Saccharofermentans* and *[Ruminococcus] gauvreauii *group, and positively related to *norank__f*__*Ruminococcaceae*. The “fluid shear stress and atherosclerosis” profile was negatively related to *Saccharofermentans*, *unclassified_f*__*Lachnospiraceae* and *[Ruminococcus] gauvreauii *group. Hence, it was concluded that *[Ruminococcus] gauvreauii *group and *norank_f*_*Ruminococcaceae* may be the core bacteria that play potential regulatory roles in the type1 and type2 enterotypes, respectively, and are worth further study.

### Rumen metabolome profiles of the two enterotypes

**Fig. 4:**
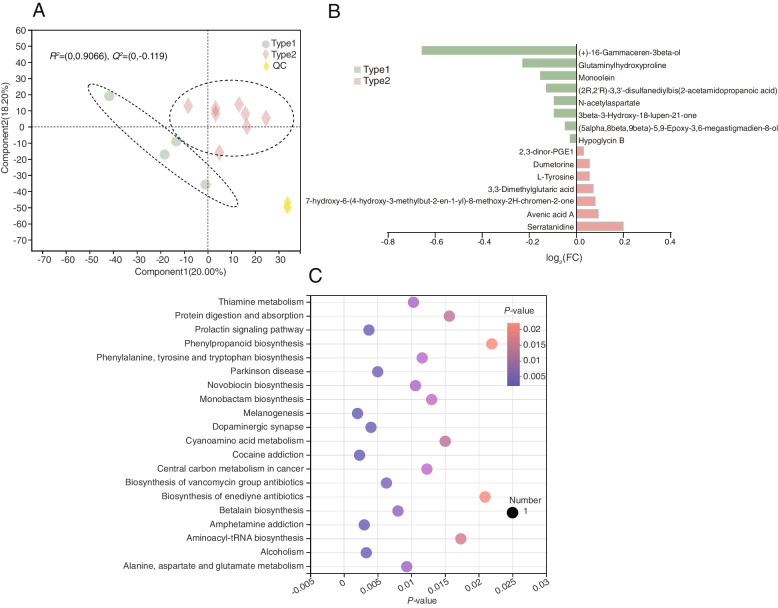
Ruminal metabolome profiles of type1 and type2 cows. **A** Ruminal metabolome profiles of type1 and type2 rumen samples based on orthogonal partial least squares discrimination analysis (OPLS-DA). **B** Ruminal differential metabolites between type1 and type2 cows using Student’s *t*-test. **C** Pathway enrichment analysis performed using the significantly different rumen metabolites between type1 and type2 cows

A total of 260 compounds were identified in the rumen metabolome. They were classified based on the two enterotypes using the PLS-DA analysis (*R*^2^*X* = 0.872, *Q*^2^*Y* = −0.263) (Fig. 4A). After performing a *t*-test with FDR < 0.05 and VIP > 1 filtering for the relative concentrations of rumen metabolites, 15 metabolites (Additional file 2: Table S5), mainly belonging to amino acids, peptides, and analogues, fatty acids and conjugates, hydroxycoumarins, pyranones and derivatives, and triterpenoids classifications, were found to be significantly changed between the two enterotypes (Fig. 4B). Metabolic pathway analysis (MetPA) based on these 15 significantly differential ruminal metabolites revealed the enrichment of 28 pathways, out of which 19 pathways were significantly different pathways (*P* < 0.05) (Fig. 4C, Additional file 2: Table S6). Notably, all these 19 pathways, namely, prolactin signaling pathway, biosynthesis of vancomycin group antibiotics, biosynthesis of enediyne antibiotics, novobiocin biosynthesis, isoquinoline alkaloid biosynthesis, thiamine metabolism, melanogenesis, methane metabolism, betalain biosynthesis, Parkinson’s disease, dopaminergic synapse, monobactam biosynthesis, alanine, aspartate and glutamate metabolism, cocaine addiction, amphetamine addiction, ubiquinone and other terpenoid-quinone biosynthesis, phenylalanine, tyrosine and tryptophan biosynthesis, and alcoholism were enriched by two metabolites, namely, *N*-acetylaspartate and *L*-tyrosine.

Considering the underlined causal relationship between ruminal metabolome and rumen microbial fermentation, a correlation analysis between ruminal differential metabolome and microbiome driven by enterotypes was performed (Additional file 1: Fig. S4). The results revealed that, among the above metabolites involved in different pathways (*N*-acetylaspartate and *L*-tyrosine), *Prevotella*, which was found in highest abundance in type1, was negatively related to *L*-tyrosine. *Ruminococcus*, which was found in highest abundance in type2, was positively related to *L*-tyrosine. Further, the hub genera of the network, *[Ruminococcus] gauvreauii *group, was negatively correlated with *L*-tyrosine, and *norank_f*_*Ruminococcaceae* was positively correlated with *L*-tyrosine (Additional file 1: Fig. S4a).

### Serum metabolome was differed between the two different enterotypes

**Fig. 5:**
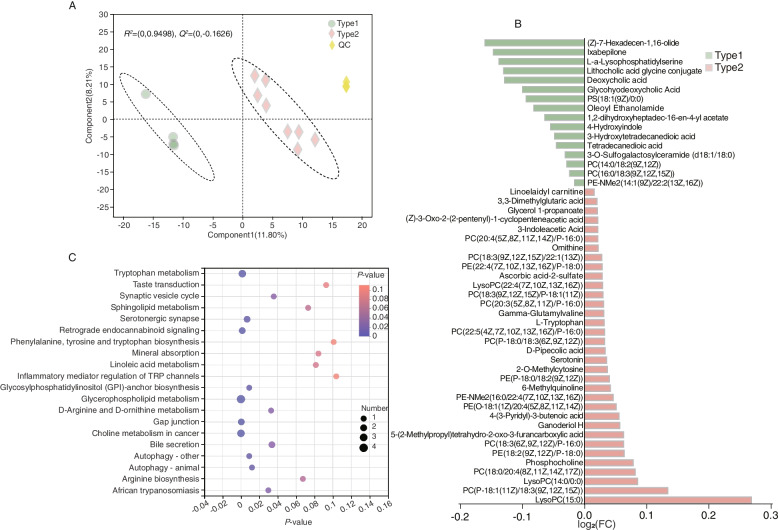
Serum metabolome profiles of type1 and type2 cows. **A** Serum metabolome profiles of type1 and type2 rumen samples based on orthogonal partial least squares discrimination analysis (OPLS-DA). **B** Serum differential metabolites between type1 and type2 cows using Student’s *t*-test. **C** Pathway enrichment analysis performed using the significantly different serum metabolites between type1 and type2 cows

A total of 113 compounds were identified in the rumen metabolome. They were classified based on the two enterotypes using the OPLS-DA analysis (*R*^2^*X* = 0.950, *Q*^2^*Y* = −0.163) (Fig. 5A). Further, 50 significantly differential metabolites, mainly belonging to “glycerophosphoethanolamines”, “glycerophosphocholines”, “amino acids, peptides, and analogues”, “bile acids, alcohols and derivatives”, “amines”, “triterpenoids”, “fatty acids and conjugates”, “fatty acyl glycosides”, “fatty acid esters”, “indolyl carboxylic acids and derivatives”, “isoflavonoid O-glycosides”, “monoradylglycerols” “cholestane steroids”, “oxosteroids”, “phosphate esters”, “terpene glycosides”, and “1-hydroxy-2-unsubstituted benzenoids” classifications were identified between the two enterotypes (Fig. 5B). MetPA based on these 50 significantly different serum metabolites (Additional file 2: Table S7) revealed the enrichment of 31 pathways, out of which 13 pathways were significantly different pathways (*P* < 0.05) (Fig. 5C, Additional file 2: Table S8). The 13 pathways, namely, choline metabolism in cancer, glycerophospholipid metabolism, *D*-arginine and *D*-ornithine metabolism, retrograde endocannabinoid signaling, tryptophan metabolism, serotonergic synapse, autophagy—other, glycosylphosphatidylinositol (GPI)-anchor biosynthesis, autophagy—animal, African trypanosomiasis, gap junction, bile secretion, and synaptic vesicle cycle were mainly enriched by PE(O-18:1(1Z)/20:4(5Z,8Z,11Z,14Z)), ornithine, phosphocholine, serotonin, *L*-tryptophan, deoxycholic acid, lysoPC(15:0), lysoPC(22:4(7Z,10Z,13Z,16Z)), PC(18:3(6Z,9Z,12Z)/P-16:0), and 3-indoleacetic acid.

Similarly, the relationships between ruminal microbiome and serum metabolome, and between ruminal and serum metabolome were also identified. The results revealed that, among the above metabolites involved in different pathways, *Prevotella* was negatively related to ornithine and lysoPC(15:0). *Ruminococcus* was positively related to ornithine, lysoPC(15:0),* L*-tryptophan, and phosphocholine. Further, the hub genera of the network, *[Ruminococcus] gauvreauii *group, was positively correlated with deoxycholic acid and negatively correlated with serotonin and lysoPC(15:0). *norank_f_Ruminococcaceae* was positively correlated with phosphocholine, serotonin, *L*-tryptophan, lysoPC(15:0), lysoPC(22:4(7Z,10Z,13Z,16Z)), and PC(18:3(6Z,9Z,12Z)/P-16:0), and negatively correlated with deoxycholic acid (Additional file 1: Fig. S4b). We also found that ruminal *N*-acetylaspartate was negatively correlated with *L*-tryptophan. Ruminal *L*-tyrosine was positively correlated with *L*-tryptophan and lysoPC(15:0), and was negatively correlated with deoxycholic acid (Additional file 1: Fig. S4C).

### Identification of the milk metabolome

**Fig. 6:**
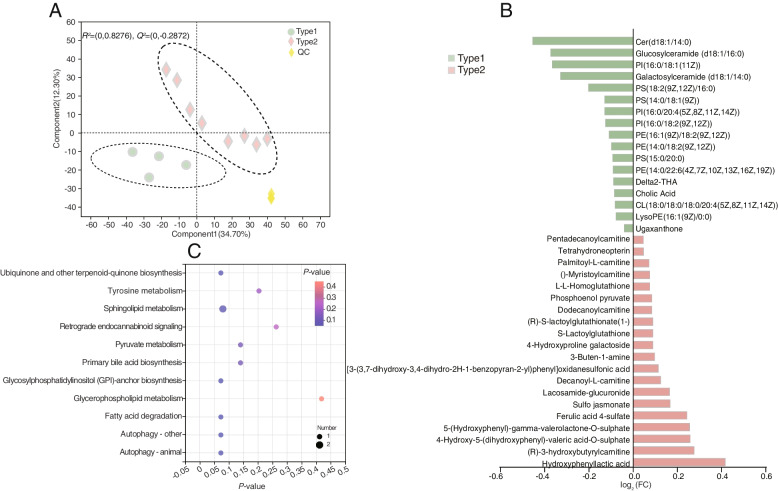
Milk metabolome profiles of type1 and type2 cows. **A** Milk metabolome profiles of type1 and type2 rumen samples based on orthogonal partial least squares discrimination analysis (OPLS-DA). **B** Milk differential metabolites between type1 and type2 cows using Student’s *t* test. **C** Pathway enrichment analysis performed using the significantly different milk metabolites between type1 and type2 cows

A total of 94 compounds were identified in the rumen metabolome. They were classified based on the two enterotypes using the OPLS-DA analysis (*R*^2^*X* = 0.828, *Q*^2^*Y* = −0.287) (Fig. 6A). After *t*-test and VIP filtering for the relative concentrations of milk metabolites, 37 significantly different metabolites (Additional file 2: Table S9), mainly belonging to “glycerophosphoinositols”, “carbohydrates and carbohydrate conjugates”, “benzoic acids and derivatives”, “fatty acid esters”, “fatty acids and conjugates”, “fatty acyl glycosides”, “pregnane steroids”, and “pyrimidines and pyrimidine derivatives”, were found to be significantly different between the two enterotypes (Fig. 6B). MetPA based on these 37 significantly different milk metabolites revealed the enrichment of 12 pathways (Fig. 6C, Additional file 2: Table S10). The 12 pathways, namely, autophagy-other, glycosylphosphatidylinositol (GPI)-anchor biosynthesis, ubiquinone and other terpenoid-quinone biosynthesis, fatty acid degradation, autophagy-animal, sphingolipid metabolism, primary bile acid biosynthesis, pyruvate metabolism, tyrosine metabolism, retrograde endocannabinoid signaling, glycerophospholipid metabolism, and bile secretion were enriched by cholic acid, PE(14:0/22:6(4Z,7Z,10Z,13Z,16Z,19Z)), tetrahydroneopterin, palmitoyl-*L*-carnitine, *S*-lactoylglutathione, glucosylceramide (d18:1/16:0), hydroxyphenyl acetic acid, and galactosylceramide (d18:1/14:0).

Further, the relationships between milk metabolome and protein composition, between ruminal microbiome and milk metabolome, between serum and milk metabolome, and between ruminal and milk metabolome were also identified. The results revealed that, among the above metabolites involved in pathways, MP and MPY were positively correlated with hydroxyphenyllactic acid, tetrahydroneopterin, and *S*-lactoylglutathione (Additional file 1: Fig. S4d). *Ruminococcus* was negatively correlated with cholic acid and PE(14:0/22:6(4Z,7Z,10Z,13Z,16Z,19Z)) (Additional file 1: Fig. S4e). Ruminal *L*-tyrosine was positively correlated with tetrahydroneopterin (Additional file 1: Fig. S4f). The serum serotonin was positively correlated with palmitoyl-*L*-carnitine and negatively correlated with glucosylceramide (d18:1/16:0), hydroxyphenyllactic acid, galactosylceramide (d18:1/14:0), and tetrahydroneopterin. The serum phosphocholine was positively correlated with cholic acid, PE(14:0/22:6(4Z,7Z,10Z,13Z,16Z,19Z)), palmitoyl-*L*-carnitine, and *S*-lactoylglutathione, and was negatively correlated with hydroxyphenyllactic acid, galactosylceramide (d18:1/14:0), and tetrahydroneopterin. The serum ornithine was positively correlated with galactosylceramide (d18:1/14:0) and negatively correlated with palmitoyl-*L*-carnitine and *S*-lactoylglutathione. The serum PE(O-18:1(1Z)/20:4(5Z,8Z,11Z,14Z)) was positively correlated with galactosylceramide (d18:1/14:0) and tetrahydroneopterin. The serum PC(18:3(9Z,12Z,15Z)/22:1(13Z)) was negatively correlated with hydroxyphenyllactic acid and tetrahydroneopterin. The serum 3-indoleacetic acid was positively correlated with glucosylceramide (d18:1/16:0). The serum lysoPC(22:4(7Z,10Z,13Z,16Z)) and lysoPC(15:0) was negatively correlated with *S*-lactoylglutathione. The serum deoxycholic acid was positively correlated with glucosylceramide (d18:1/16:0), hydroxyphenyllactic acid, galactosylceramide (d18:1/14:0) and tetrahydroneopterin. The serum *L*-tryptophan was positively correlated with galactosylceramide (d18:1/14:0) (Additional file 1: Fig. S4g).

### Explanation of pathways established based on the relationships among the ruminal microbiome and metabolome, and serum metabolome and milk metabolome, to the MPY

**Fig. 7:**
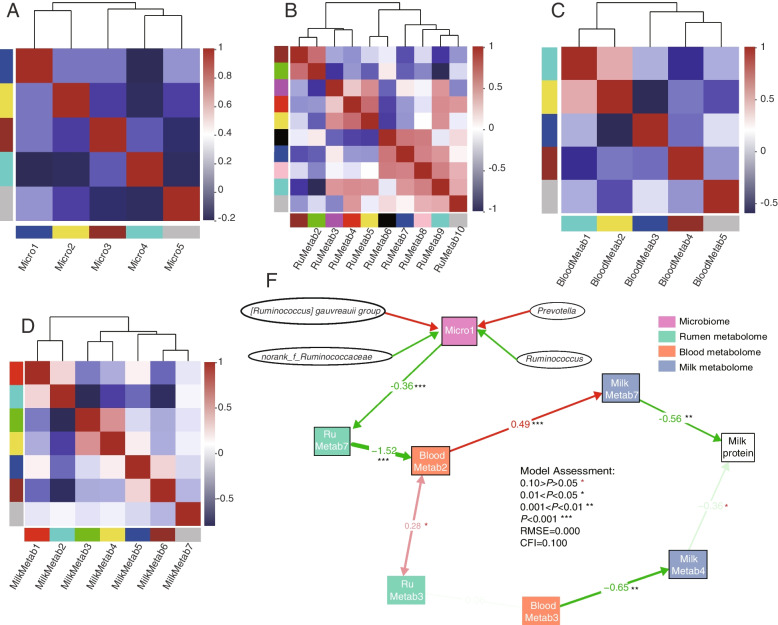
WGCNA and SEM of rumen bacteria. **A** Relationship of modules among microbial abundance at the genera level (modules are named by colors). **B** Relationship of modules among ruminal metabolites at the genera level (modules are named by colors). **C** Relationship of serum metabolites at the genera level (modules are named by colors). **D** SEM was established using milk protein and the modules of rumen microbiome, rumen metabolome, serum metabolome, and milk metabolome in the WGCNA analysis. **E** Numbers adjacent to arrows are indicative of the effect size of the relationship. R^2^ denotes the proportion of variance explained. Red arrows represent positive paths and green arrows represent negative paths. Significance levels are as follows: ^*^P < 0.05; ^**^P < 0.01; ^***^P < 0.001. RMSEA, root mean square error of approximation; CFI, comparative fit index

The SEM based on the WGCNA analysis was established to link the different modules of each omics based on the logic of “rumen-serum-milk-MPY”. For WGCNA analysis, the rumen microbiome was divided into five microbial modules (Fig. 7A and Additional file 2: Table S11). Micro1 included *Prevotella* and *[Ruminococcus] gauvreauii *group (which drives type1), and *Ruminococcus* and *norank_f*_*Ruminococcaceae* (which drives type2). Moreover, the rumen, serum, and milk metabolomes were divided into 10, 5, and 7 metabolomic modules respectively (Fig. 7B–D and Additional file 2: Table S12–14).

Next, in order to explore the relationship between multiple omics, the SEM was established to analyse the core metabolomic module based on the expression level of the modules and their relationships (Additional file 1: Fig. S5A and Additional file 2: Table S15). Finally, a metabolic pathway from micro1 to MPY was established (RMSE = 0.000, CFI = 1.000; Fig. 7E). The metabolic pathway was “micro1-rumentab7-bloodmetab2-milkmetab7-MPY”. For micro1, the correlation analysis showed that *Prevotella* and *[Ruminococcus] gauvreauii *group positively regulated micro1, *Ruminococcus* and *norank_f*_*Ruminococcaceae* negatively regulated micro1 (Additional file 1: Fig. S5B). Moreover, *[Ruminococcus] gauvreauii *group and *norank_f*_*Ruminococcaceae*, but not *Prevotella* and *Ruminococcus*, were found to be the hub genera in the network of micro1 (Additional file 1: Fig. S6A and Additional file 2: Table S16). The function of micro1 mainly included “biosynthesis of amino acids”, “purine metabolism”, “carbon metabolism”, “ABC transports” and “starch and sucrose metabolism” (TOP5) (Additional file 1: Fig. S6B). For rumetab7 (Additional file 1: Fig. S7A), the metabolites mainly belonged to “sesquiterpenoids”, “amino acids, peptides, and analogues” and “carbohydrates and carbohydrate conjugates” (TOP3). These metabolites were mainly enriched in “tyramine metabolites”, “toluene degradation”, “protein digestion and absorption”, “prolactin signaling pathway” and so on. For bloodmetab2 (Additional file 1: Fig. S7B), the metabolites mainly belonged to “lipids and lipid-like molecules”, “organic acids and derivatives” and “phenylpropanoids and polyketides” (TOP3). These metabolites were mainly enriched in “choline metabolism in cancer”, “autophagy-animal”, “protein digestion and absorption”, “glycerophospholipid metabolism” and so on. For milkmetab7 (Additional file 1: Fig. S7C), the metabolites mainly belonged to “glycerophosphoinositols” and “carbohydrates and carbohydrate conjugates” (TOP3). These metabolites were mainly enriched in “prolactin signaling pathway”, “steroid hormone biosynthesis”, “aldosterone synthesis and secretion”, and “pathways in cancer”.

**Fig. 8:**
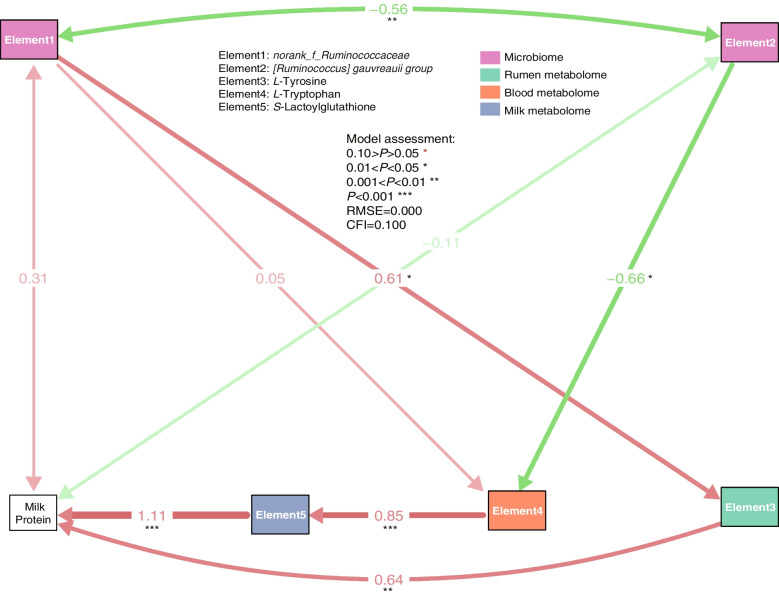
SEM established by the *Ruminococcus_gauvreauii*, *norank_f_Ruminococcus*, tyrosine, tryptophan, *S*-lactoylglutathione, and milk protein. Numbers adjacent to arrows are indicative of the effect size of the relationship. R^2^ denotes the proportion of variance explained. Red arrows represent positive paths and green arrows represent negative paths. Significance levels are as follows: ^*^P < 0.05; ^**^P < 0.01; ^***^P < 0.001. RMSEA, root mean square error of approximation; CFI, comparative fit index

Combined with the SEM established by the modules and the identified differential metabolites and enriched metabolome pathways, the *L*-tyrosine in the rumetab7 and *L*-tryptophan in the bloodmetab2 and their potential roles in regulating the MPY were studied. To do this, we established the SEM based on the* L*-tyrosine of rumen and *L*-tryptophan in the blood (Fig. 8 and Additional file 1: Fig. S8). *[Ruminococcus] gauvreauii *group and *norank_f*_*Ruminococcaceae* could establish the module with high fitness, which indicated that these two genera can regulate the milk protein yield by affecting the *L*-tyrosine and *L*-tryptophan biosynthesis (RMSE = 0.000, CFI = 1.000). On the other hand, the module established by *Ruminococcus* and *Prevotella* had poor fitness (RMSE = 0.500, CFI = 0.741).

## Discussion

By integrating ruminal microbiome and metabolome, serum metabolome, and milk metabolome, we investigated the effect of rumen enterotypes on lactation performance. The results suggested that the enterotypes could affect the microbial metabolome (rumen) and host metabolome (serum and milk), which further led to differences in MPY biosynthesis. In a previous study, bacteria had been proved to serve as the most important contributors to MPY, when compared to other microbial kingdoms [3]. Hence, we focused on the rumen bacterial enterotypes based on 16S rRNA gene amplicon sequencing, with an aim to identify key bacteria and link the key bacteria driven ruminal microbiome to the milk protein synthesis ability.

In this study, two enterotypes were identified from 12 dairy cows —type1 driven by *Prevotella* and type2 driven by *Ruminococcus*. These two enterotypes are most common in gastrointestinal microbiological research. The enterotypes could be prominently found in the key microbiota associated with phenotype [36]. In wild fauna and humans, gastrointestinal microorganisms may not obviously cluster to be explained by enterotypes, because of the influence of external environmental factors, which cannot be avoided [12]. But in the case of domestic animals, enterotypes analysis can be more advantageous, as the physiological state, diet and environment can be controlled. In this study, we found the key microbiota regulating MPY in dairy cows, using enterotype analysis. The enterotype driven by *Prevotella* was associated with the degradation of structural carbohydrate (e.g., fibre). However, the enterotype driven by *Ruminococcus* was associated with the degradation of non-structural carbohydrate (e.g., starch) [7]. It is to be noted that *Prevotella* was often positively related with amino acid metabolism, especially for branched-chain amino acids (BCAA) [37]. More importantly, *Prevotella* was an important contributor to the precursor of milk protein synthesis in dairy cows [3]. In this study, the MPY of type2 enterotypes was higher than the MPY of type1 enterotypes. In type1, the relative abundance of *Prevotella* and *Ruminococcus* was 36.76% and 4.74%, respectively. In type2, the relative abundance of *Prevotella* and *Ruminococcus* was 22.46% and 24.67%, respectively. Hence, the high MPY could not be attributed just to *Prevotella*; the synthesis of microbial protein was also needed for energy supply. *Ruminococcus* played an important role in releasing energy through the degradation of high grain diet (high starch) [38]. The relative abundance of *Ruminococcus* was close to *Prevotella* in type2, which is more consistent with the rumen energy-nitrogen balance principle [39]. Hence, rumen microbiota driven by *Ruminococcus* may improve the synthesis of microbial protein by creating a better balance between energy and nitrogen in rumen, which further increased the MPY of dairy cows. Here, we also inferred that the rumen energy-nitrogen balance principle not only considered the energy degradation rate and protein degradation rate of feed raw materials [40], but also ensured the balance of rumen microbial community, for example, the ratio between *Prevotella* and *Ruminococcus*.

The enterotypes altered the rumen, serum, and milk metabolite compositions. In the rumen, *L*-tyrosine was increased in type2. *L*-tyrosine treatment could increase the milk yield, milk protein and conception rate in dairy cows during the early lactation period [41]. Tyrosine has had a wide range of effects on lactation in dairy cows. Tyrosine is an important precursor of neurotransmitter synthesis, such as catecholamine, which could increase the energy intake of mammary gland cells by activating growth hormones [42, 43]. Tyrosine is also an important source of casein, which is the main component of milk protein [44]. Moreover, tyrosine is also an important raw material for bacteria to synthesize thiamine, which could stabilize the bacterial community as well as increase the pH of rumen [45]. Notably, thiamine metabolism of rumen microbiota could help host tolerant high grain diet [46, 47], which could produce more energy for lactation. In the blood, the type2 enterotypes had higher *L*-tryptophan, 3-indoleacetic acid and serotonin, which facilitate stronger tryptophan metabolism. Rumen-protected tryptophan supplementation can increase lactation performance [48]. Serotonin can regulate maternal and mammary calcium homeostasis through a serotonin-calcium feedback loop involving endocrine and autocrine/paracrine [49]. More importantly, serotonin could increase feed intake [50], which is closely related to lactation of dairy cows. Moreover, the type2 enterotypes had higher serum ornithine, which indicates a stronger urea cycle of the body [51]. For ruminants, the urea cycle could provide more urea nitrogen for rumen microbial protein [52]. In the milk, increased palmitoyl-*L*-carnitine in the type2 enterotypes could promote the energy supply of fatty acid oxidation via the transportation of long-chain fatty acids to ATP [53]. Increased *S*-lactoylglutathione in the type2 enterotypes is oxidized to pyruvate via *D*-lactate dehydrogenase and as a consequence, electrons flow to oxygen, producing energy and ATP synthesis [54]. Moreover, increased tetrahydroneopterin in the type2 enterotypes was an essential cofactor for tyrosine metabolism and tryptophan metabolism. Tetrahydroneopterin was also an obligate cofactor of nitric oxide (NO) synthases. For mammary, the NO could regulate milk compositions transport by controlling the mammary blood flow [55]. Thus, increased metabolites of rumen, serum, and milk could provide the raw materials and energy for milk protein synthesis.

Host traits, including methane production [4], feed efficiency [5], and milking traits [56] were attained as a result of the crosstalk between rumen microbiota and the host. Hence, we focused not only on the rumen microbial metabolome, but also on the serum and milk metabolome. However, the relationship between microbial composition and metabolism with host metabolism was not thoroughly studied. Hence, the conjoint analysis of WGCNA and SEM combined with the rumen microbial metabolism and host metabolism was done to explain the MPY. Firstly, the rumen microbiome was divided into five modules, based on the WGCNA analysis. Out of these modules, micro1 included enterotype-driving bacteria such as *Prevotella*, *[Ruminococcus] gauvreauii *group, *Ruminococcus* and *norank_f*_*Ruminococcaceae*, which suggested that the microbiota of micro1 may be the core microbiota driven by the enterotypes. Hence, the SEM was used to untangle the metabolic pathway from micro1 to MPY. Here, we think that the SEM could find the host metabolic modules that were not directly regulated by the rumen microbiota. Finally, we concluded that rumetab7, bloodmetab2, and milkmetab7 may be the key modules responsible for the regulation of MPY by micro1. In the SEM established by the modules of WGCNA, the significantly increased tyrosine and tryptophan were clustered to rumetab7 and bloodmetab2, respectively. In order to clarify the relationship between omics, the SEM was established by *Prevotella*, *[Ruminococcus] gauvreauii *group, *norank_f*_*Ruminococcaceae*, tyrosine, and tryptophan. The SEM module with high fitness suggested that the *norank_f*_*Ruminococcaceae* of type1 could increase the rumen tyrosine, which provides the substrate and energy for milk protein synthesis. The tryptophan metabolism (eg., melatonin and serotonin) was found to enhance the glyoxalase system [57, 58]. *S*-lactoylglutathione, which is an intermediate of the glyoxalase system, could provide energy for milk protein synthesis [59]. *[Ruminococcus] gauvreauii *group in the type1 enterotypes could inhibit the regulation of tryptophan on milk protein synthesis. Interestingly, *Prevotella* and *Ruminococcus* could not derive the metabolite to regulate milk protein synthesis in the SEM module (Additional file 1: Fig. S8). But they could derive the micro1 to regulate milk protein synthesis, which suggested that the *Prevotella* and *Ruminococcus* may not function alone.

There are several limitations in the present study. First, our study provides evidence that ruminal enterotypes, especially *Ruminococcus*, which may act with the other ruminal bacteria, can regulate the MPY by affecting the ruminal tyrosine. This result is logically reliable and can provide a novel insight to link ruminal microbiota with the MPY, which was worthy to validate in a larger cohort. Furthermore, although the host metabolome could reflect the host genetics information to some degree, except for the ruminal microbiota and metabolome, and the host metabolome. Study of the interaction between host genetics and microbiome that contributed to the MPY is still lacking. Hence, additional studies of a larger cohort, focusing on the interaction between host genetics and ruminal metagenome changes and their contribution to the MPY are worth performing.

## Conclusions

Taken together, based on the enterotype analysis, the joint analysis of multi-omics based on the WGCNA and SEM suggest that the represented enterotype genera of *Prevotella* and *Ruminococcus*, and the hub genera of *[Ruminococcus] gauvreauii *group and *norank_f*_*Ruminococcaceae* could regulate milk protein synthesis. Rumen tyrosine and serum tryptophan play an important role in the path analysis of the structural equation model. The structural equation model established by metabolites suggested that *norank_f*_*Ruminococcaceae*, not *Ruminococcus* could increase the rumen tyrosine, which provides the substrate for milk protein synthesis. *[Ruminococcus] gauvreauii *group, not *Prevotella* could inhibit serum tryptophan by providing pyruvate metabolic raw material (*S*-lactoylglutathione) for the mammary gland. In summary, the study achieved joint analysis of multi-omics through weighted gene co-expression network analysis and structural equation model, which provide new insights into host-microbiota crosstalk for milk protein synthesis in dairy cows.

## Supplementary Information

**Additional file 1: Fig. S1.** Tests to determine the optimal soft threshold power for WGCNA. Tests to determine the optimal soft threshold power for rumen microbiome modules at the genera level (**A**), rumen metabolome (**B**), serum metabolome (**C**), and milk metabolome (**D**). **Fig. S2.** The relative abundance of rumen bacteria of 12 cows at the genera level. **Fig. S3.** The network analysis for ruminal enterotypes. **A** The network analysis of type1. **B** The network analysis of type2. **Fig. S4.** The correlation analysis between different bacteria and metabolites between enterotypes of rumen microbiome, rumen metabolome, serum metabolome, milk metabolome, and milk protein. **Fig. S5.** The correlation analysis between modules of WGCNA and differential bacteria of enterotypes. **A** The correlation analysis between modules of rumen microbiome, rumen metabolome, serum metabolome, and milk metabolome in the WGCNA analysis. **B** The correlation analysis between modules of rumen microbiome, rumen metabolome, serum metabolome, and milk metabolome with different bacteria of enterotypes. **Fig. S6.** The microbial compositions and functions profiles of micro1 module of WGCNA. **A** The network analysis of micro1 using 16S rRNA sequence data. **B** The function of micro1 using metagenome data. **Fig. S7.** The metabolome profiles of rumetab7, bloodmetab2, and milkmetab7 module. **A** Classification of metabolic compounds based on the Human Metabolome Database (HMDB). **B** Pathway enrichment analysis. **Fig. S8.** The SEM established by the Pervotella, Ruminococcus, tyrosine, tryptophan, *S*-lactoylglutathione, and milk protein. Numbers adjacent to arrows are indicative of the effect size of the relationship. *R*^2^ denotes the proportion of variance explained. Red arrows represent positive paths and green arrows represent negative paths. Significance levels are as follows: ^*^ *P* < 0.05; ^**^P < 0.01; ^***^P < 0.001. RMSEA, root mean square error of approximation; CFI, comparative fit index.

**Additional file 2: Table S1.** The differential rumen bacteria between type1 and type2. **Table S2.** The evaluation of network established by all samples. **Table S3.** The evaluation of network established by type1. **Table S4.** The evaluation of network established by type2. **Table S5.** The differential rumen metabolites between type1 and type2. **Table S6.** The enriched KEGG pathways of differential rumen metabolites between type1 and type2. **Table S7.** The differential serum metabolites between type1 and type2. **Table S8.** The enriched KEGG pathways of serum rumen metabolites between type1 and type2. **Table S9.** The differential milk metabolites between type1 and type2. **Table S10.** The enriched KEGG pathways of milk rumen metabolites between type1 and type2. **Table S11.** The bacteria of 5 modules of rumen microbiome based on WGCNA. **Table S12.** The bacteria of 10 modules of rumen metabolome based on WGCNA. **Table S13.** The bacteria of 5 modules of serum metabolome based on WGCNA. **Table S14.** The bacteria of 7 modules of milk metabolome based on WGCNA. **Table S15.** The expression level of all modules based on WGCNA of rumen microbiome and metabolome, serum metabolome, and milk metabolome. **Table S16.** The evaluation of network established by micro1. **Table S17.** The ASVs sequence of *norank_f_Ruminococcaceae* in this study.

## Data Availability

All the data generated or analysed in this study are included in this paper. The sequencing reads of 16S rRNA gene sequencing and shotgun metagenome sequencing are both available in the Sequence Read Archive (SRA) of NCBI with PRJNA592280.

## Abbreviations

ASVs: Amplicon sequence variants
CFI: Comparative Fit Index
GLU: Glucose
MetPA: Metabolic pathway analysis
MFY: Milk fat yield
MPY: Milk protein yield
MY: Milk yield
NO: Nitric oxide
PCA: Principal component analysis
RMSE: Root mean square error
SEM: Structural equation model
TC: Total cholesterol
TG: Triglyceride
TP: Total protein
VFA: Volatile fatty acids
VIP: Projection
WGCNA: Weighted gene co-expression network
