# Supplementary material for: Multi-omics reveals that the host-microbiome metabolism crosstalk of differential rumen bacterial enterotypes can regulate the milk protein synthesis of dairy cows
Source: J Anim Sci Biotechnol. 2023 May 9;14:63. doi: 10.1186/s40104-023-00862-z (PMC10169493; doi:10.1186/s40104-023-00862-z)
Supplement: Supplementary file 1 — Additional file 1: Fig. S1. Tests to determine the optimal soft threshold power for WGCNA. Tests to determine the optimal soft threshold power for rumen microbiome modules at the genera level (A), rumen metabolome (B), serum metabolome (C), and milk metabolome (D). Fig. S2. The relative abundance of rumen bacteria of 12 cows at the genera level. Fig. S3. The network analysis for ruminal enterotypes. A The network analysis of type1. B The network analysis of type2. Fig. S4. The correlation analysis between different bacteria and metabolites between enterotypes of rumen microbiome, rumen metabolome, serum metabolome, milk metabolome, and milk protein. Fig. S5. The correlation analysis between modules of WGCNA and differential bacteria of enterotypes. A The correlation analysis between modules of rumen microbiome, rumen metabolome, serum metabolome, and milk metabolome in the WGCNA analysis. B The correlation analysis between modules of rumen microbiome, rumen metabolome, serum metabolome, and milk metabolome with different bacteria of enterotypes. Fig. S6. The microbial compositions and functions profiles of micro1 module of WGCNA. A The network analysis of micro1 using 16S rRNA sequence data. B The function of micro1 using metagenome data. Fig. S7. The metabolome profiles of rumetab7, bloodmetab2, and milkmetab7 module. A Classification of metabolic compounds based on the Human Metabolome Database (HMDB). B Pathway enrichment analysis. Fig. S8. The SEM established by the Pervotella, Ruminococcus, tyrosine, tryptophan, S-lactoylglutathione, and milk protein. Numbers adjacent to arrows are indicative of the effect size of the relationship. R2 denotes the proportion of variance explained. Red arrows represent positive paths and green arrows represent negative paths. Significance levels are as follows: * P < 0.05; **P < 0.01; ***P < 0.001. RMSEA, root mean square error of approximation; CFI, comparative fit index. [file 40104_2023_862_MOESM1_ESM.docx]

**Additional file 1**


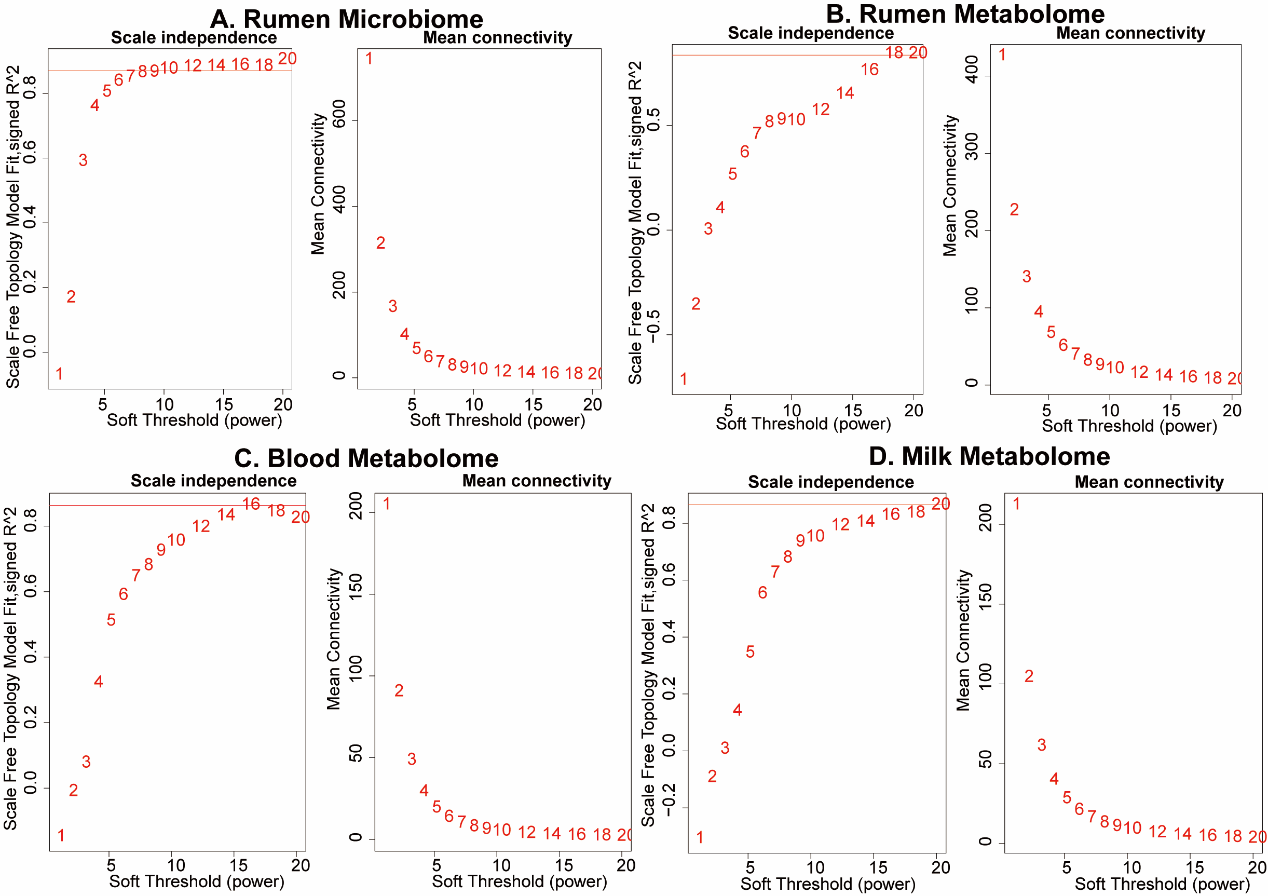


**Fig. S1** Tests to determine the optimal soft threshold power for WGCNA. Tests to determine the optimal soft threshold power for rumen microbiome modules at the genera level (**A**), rumen metabolome (**B**), serum metabolome (**C**), and milk metabolome (**D**)


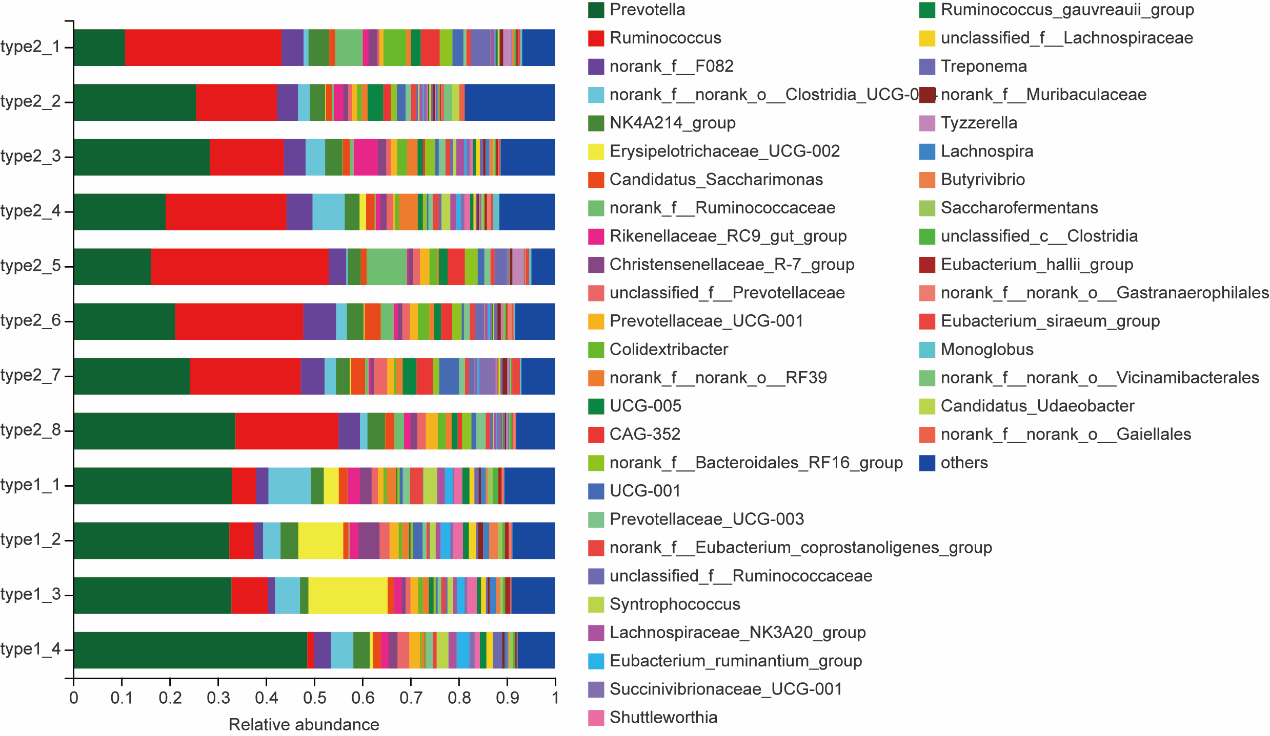


**Fig. S2** The relative abundance of rumen bacteria of 12 cows at the genera level


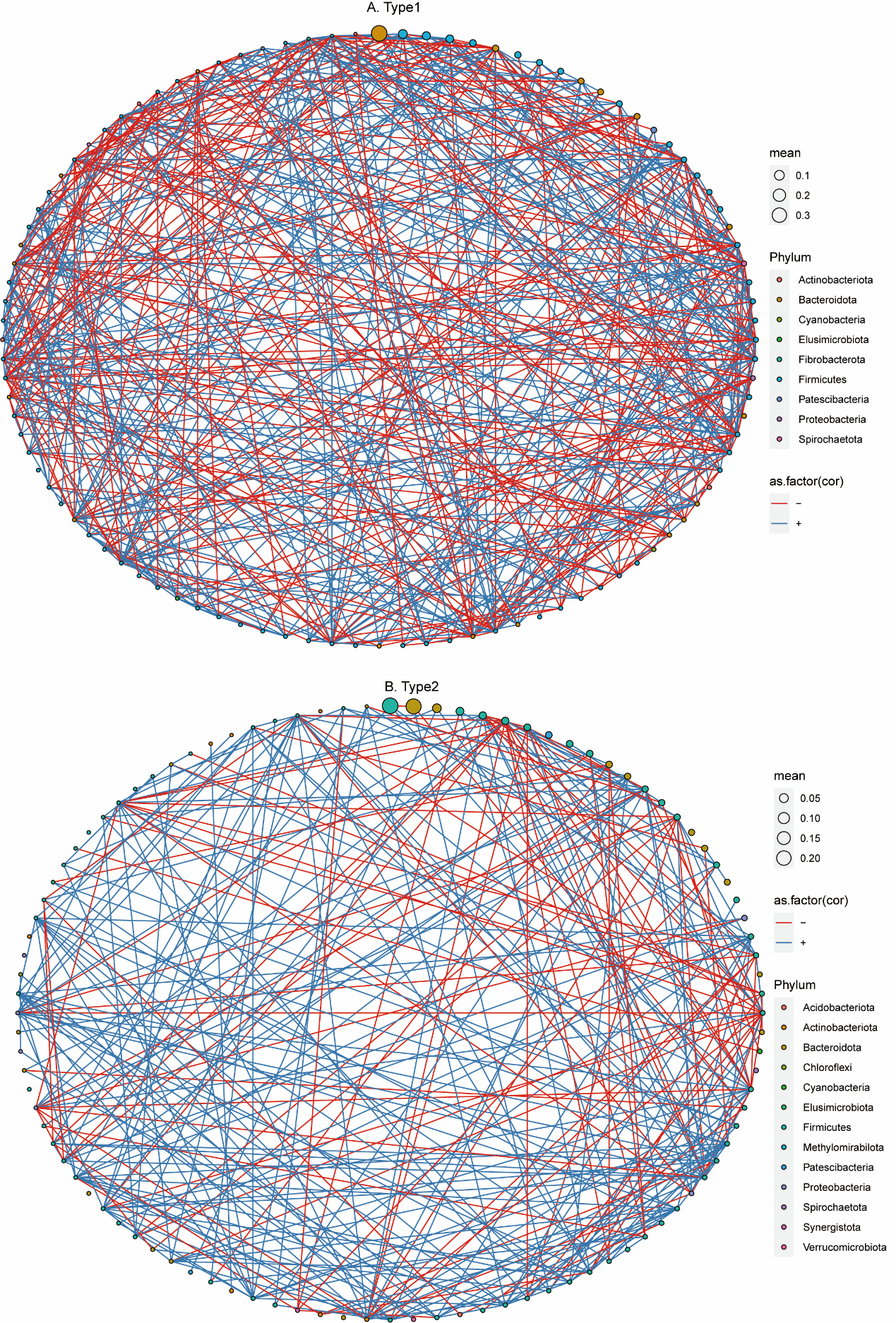


**Fig. S3** The network analysis for ruminal enterotypes. **A** The network analysis of type1. **B** The network analysis of type2


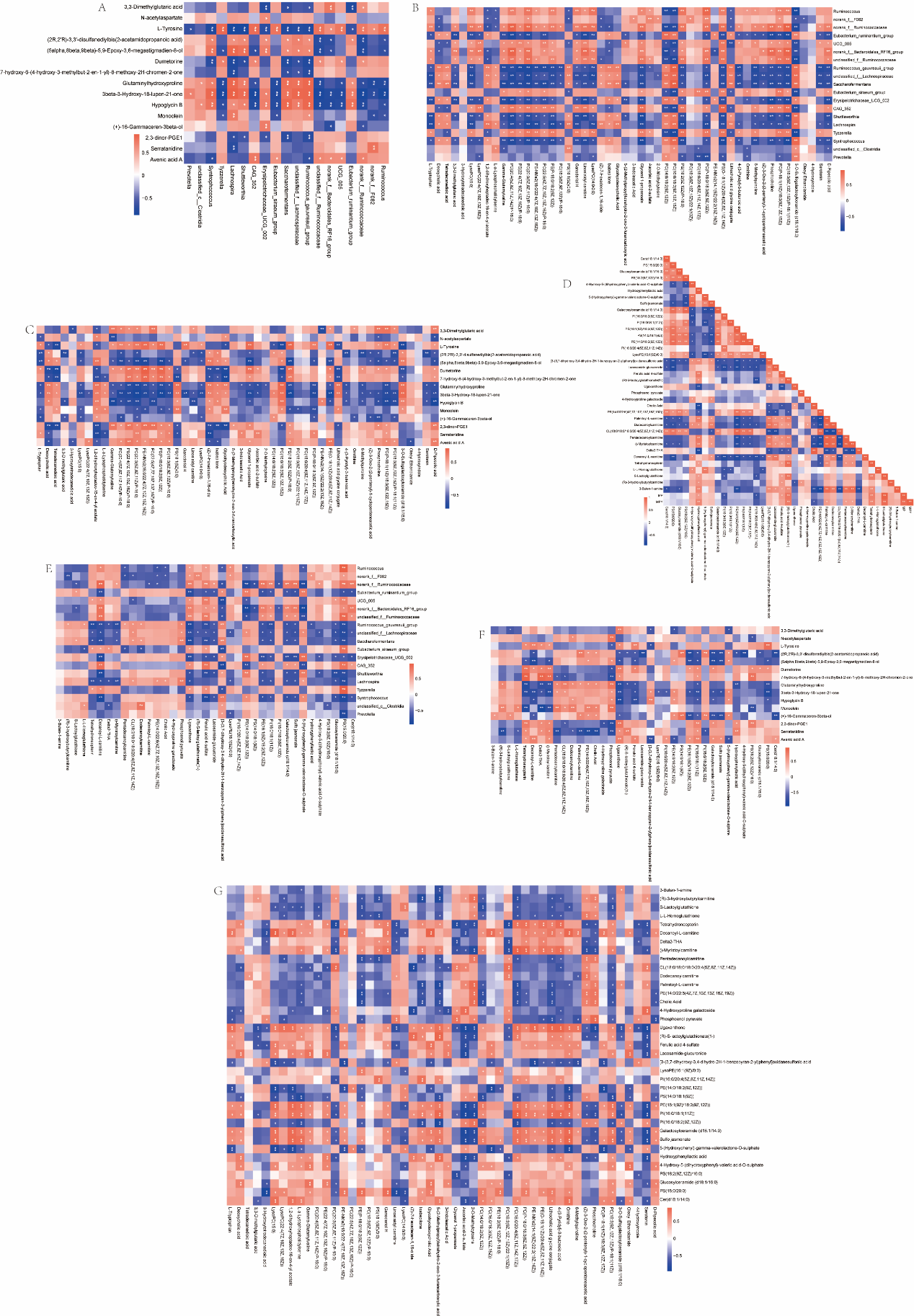


**Fig. S4** The correlation analysis between different bacteria and metabolites between enterotypes of rumen microbiome, rumen metabolome, serum metabolome, milk metabolome, and milk protein


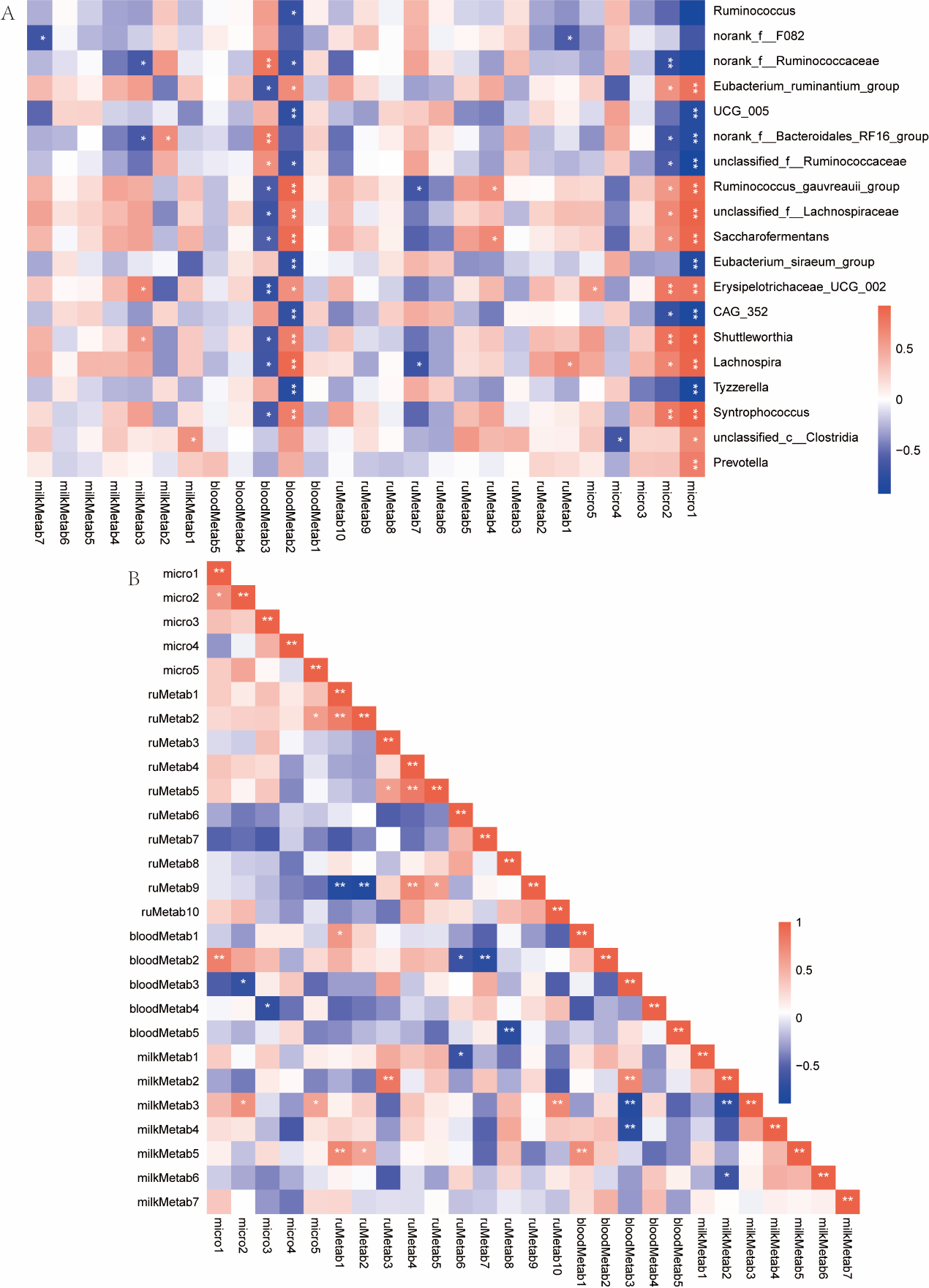


**Fig. S5** The correlation analysis between modules of WGCNA and differential bacteria of enterotypes. **A** The correlation analysis between modules of rumen microbiome, rumen metabolome, serum metabolome, and milk metabolome in the WGCNA analysis. **B** The correlation analysis between modules of rumen microbiome, rumen metabolome, serum metabolome, and milk metabolome with different bacteria of enterotypes


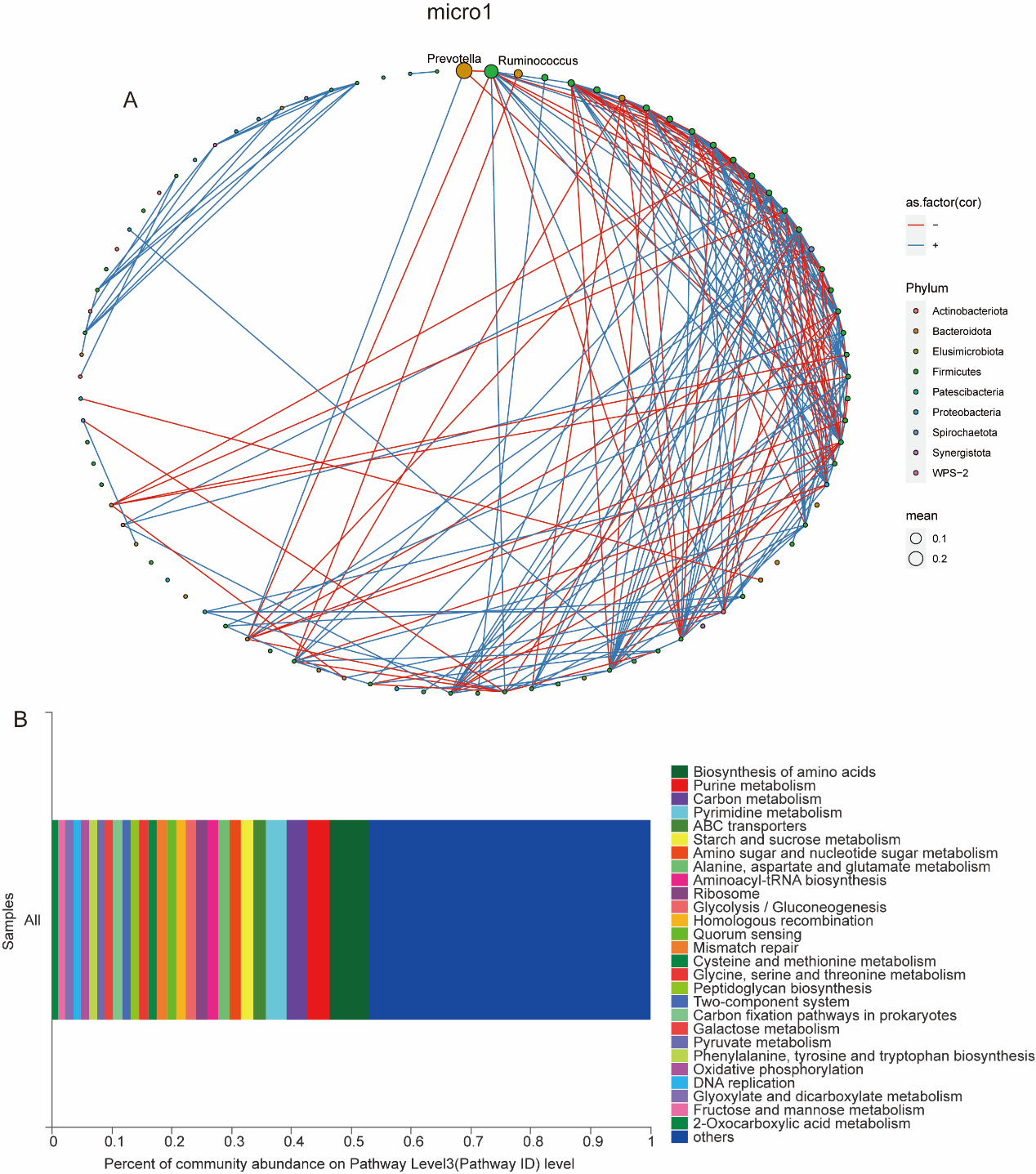


**Fig S6** The microbial compositions and functions profiles of micro1 module of WGCNA. **A** The network analysis of micro1 using 16S rRNA sequence data. **B** The function of micro1 using metagenome data


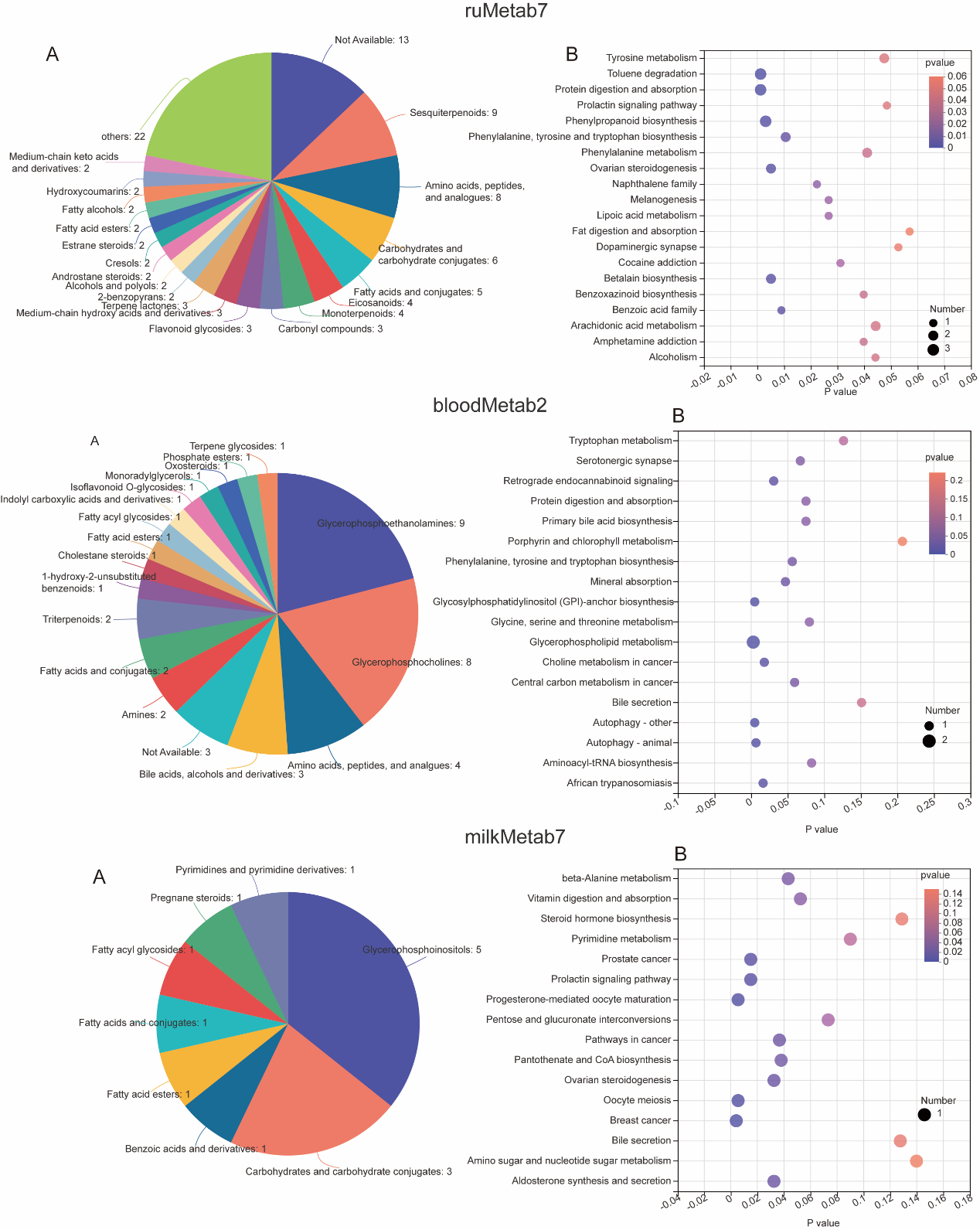


**Fig. S7** The metabolome profiles of rumetab7, bloodmetab2, and milkmetab7 module. **A** Classification of metabolic compounds based on the Human Metabolome Database (HMDB). **B** Pathway enrichment analysis


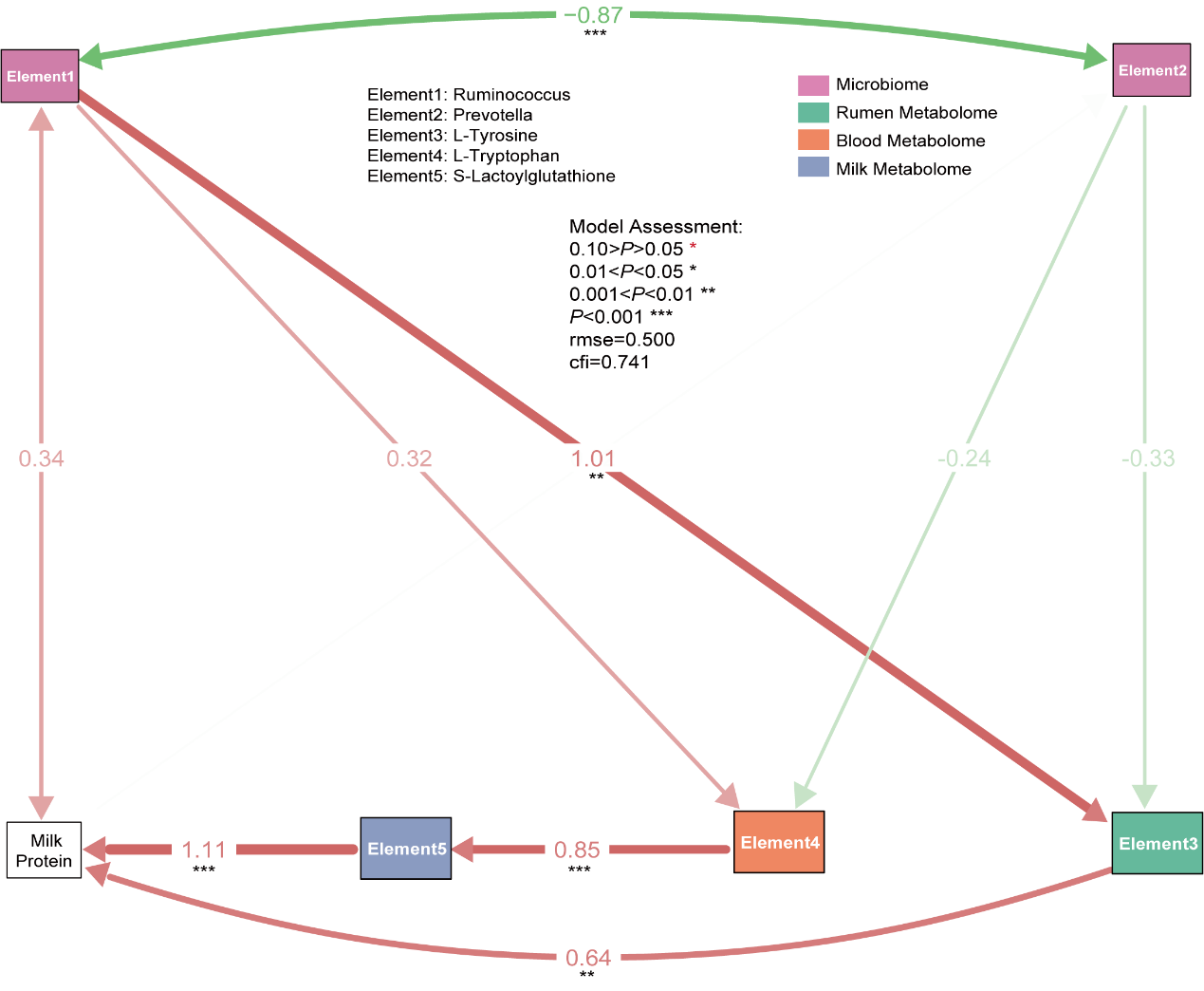


**Fig S8** The SEM established by the Pervotella, Ruminococcus, tyrosine, tryptophan, S-lactoylglutathione, and milk protein. Numbers adjacent to arrows are indicative of the effect size of the relationship. *R*^2^ denotes the proportion of variance explained. Red arrows represent positive paths and green arrows represent negative paths. Significance levels are as follows: ^*^*P* < 0.05; ^**^*P* < 0.01; ^***^*P* < 0.001. RMSEA, root mean square error of approximation; CFI, comparative fit index
